# PFKFB3 overexpression in monocytes of patients with colon but not rectal cancer programs pro-tumor macrophages and is indicative for higher risk of tumor relapse

**DOI:** 10.3389/fimmu.2022.1080501

**Published:** 2023-01-17

**Authors:** Irina Larionova, Marina Patysheva, Pavel Iamshchikov, Elena Kazakova, Anna Kazakova, Militsa Rakina, Evgeniya Grigoryeva, Anna Tarasova, Sergei Afanasiev, Natalia Bezgodova, Artem Kiselev, Alexey Dobrodeev, Dmitriy Kostromitskiy, Nadezhda Cherdyntseva, Julia Kzhyshkowska

**Affiliations:** ^1^ Laboratory of translational cellular and molecular biomedicine, National Research Tomsk State University, Tomsk, Russia; ^2^ Cancer Research Institute, Tomsk National Research Medical Center, Russian Academy of Sciences, Tomsk, Russia; ^3^ Laboratory of Genetic Technologies, Siberian State Medical University, Tomsk, Russia; ^4^ Institute for Quantitative Health Science and Engineering (IQ), Michigan State University, East Lansing, MI, United States; ^5^ Institute of Transfusion Medicine and Immunology, Institute for Innate Immunoscience (MI3), Medical Faculty Mannheim, University of Heidelberg, Mannheim, Germany; ^6^ German Red Cross Blood Service Baden-Württemberg – Hessen, Mannheim, Germany

**Keywords:** monocyte, PFKFB3, colorectal cancer, tumor-associated macrophage, transcriptome, GeoMx DSP, chemotherapy

## Abstract

**Introduction:**

Circulating monocytes are main source for tumor-associated macrophages (TAMs) that control tumor growth, angiogenesis, metastasis and therapy resistance. We raised the questions how monocyte programming is affected by growing tumors localized in colon and rectal sections, and how treatment onsets affect monocyte programming in the circulation.

**Methods:**

Patients with rectal cancer and colon cancer were enrolled in the study. Peripheral blood monocytes were characterized by phenotypic analysis using flow cytometry, by transcriptomic analysis using RNA sequencing and by gene expression analysis using real-time RT-PCR. Phenotypic analysis was performed with IF/confocal microscopy. Spatial transcriptomic analysis was applied using GeoMX DSP-NGS.

**Results:**

In patients with rectal cancer, increased amount of CCR2+ monocytes was indicative for the absence of both lymphatic and hematogenous metastasis. In contrast, in patients with colon cancer CD163+ monocytes were indicative for LN metastasis. NGS analysis identified tumor-specific transcriptional programming of monocytes in all CRC patients compared to healthy individuals. The key transcriptional difference between monocytes of patients with colon and rectal cancer was increased expression of PFKFB3, activator of glycolysis that is currently considered as therapy target for major solid cancers. PFKFB3-expressing monocyte-derived macrophages massively infiltrated tumor in colon. Nanostring technology identified correlation of PFKFB3 with amount and tumor-promoting properties of TAMs in colon but not in rectal cancer. PFKFB3 was indicative for tumor relapse specifically in colon cancer.

**Discussion:**

Our findings provide essential argument towards CRC definition to cover two clinically distinct cancers – colon cancer and rectal cancer, that differentially interact with innate immunity.

## 1 Introduction

Colorectal cancer (CRC) is third most commonly diagnosed malignancy and the third leading cause of cancer-related deaths in the world. In 2020 more than one million new cases of colorectal cancer were diagnosed, and almost 570 thousand deaths were registered worldwide (1). Colorectal cancer is characterized by high inter- and intra-tumoral heterogeneity (2). This heterogeneity is an obstacle to reach a complete response after anti-cancer therapy that requires personification (3). Although colon cancer (CC) and rectal cancer (RC) are usually classified as colorectal cancer (CRC), evidences accumulated towards considering CC and RC as self-standing tumor entities due to their topography, surgical challenge, therapy, complications, and relapse patterns (3, 4).

The key innate immune cells in tumor microenvironment (TME) are tumor-associated macrophages (TAMs) (5, 6). There are experimental and clinical data showing that TAMs promote primary tumor growth and dissemination in major cancer types, including breast, lung, kidney, ovarian, prostate cancers, melanoma, and glioblastoma (6, 7). However, in CRC the role of TAMs remains controversial. Accumulating findings indicate antitumor effect of TAMs in the number of cohorts of CRC patients (6, 8–10). Pro-tumor functions have been reported for specific TAM subpopulations expressing M2 markers CD206, CD163 and CD204 (11, 12). TAMs develop their functional phenotype in response to local TME, and are very plastic cells that can define patients’ outcome (13).

Essential factor that defines functional state of TAMs in the tumor site is the activation status of blood monocytes, which are recruited to tumor site from the circulation and give origin to TAMs (14). Circulating monocytes are also heterogeneous. Based on their phenotype and function, human monocytes can be divided into three major subsets: classical (CD14++16-), intermediate (CD14+16+) and non-classical (CD14+16++) monocytes (15). We and others found that pathological stimuli can drive changes in the activation level of monocytes affecting their potential to differentiate into functionally distinct macrophages in the tissues (16–19). Changes in monocyte activation status in pathology can serve as a protective mechanism of the organism, can be a part of the disease pathogenesis, or can reflect a response of immune system to the anti-cancer treatment (20). However, very limited knowledge is available about the cancer-specific programming of monocytes.

**Figure 1:**
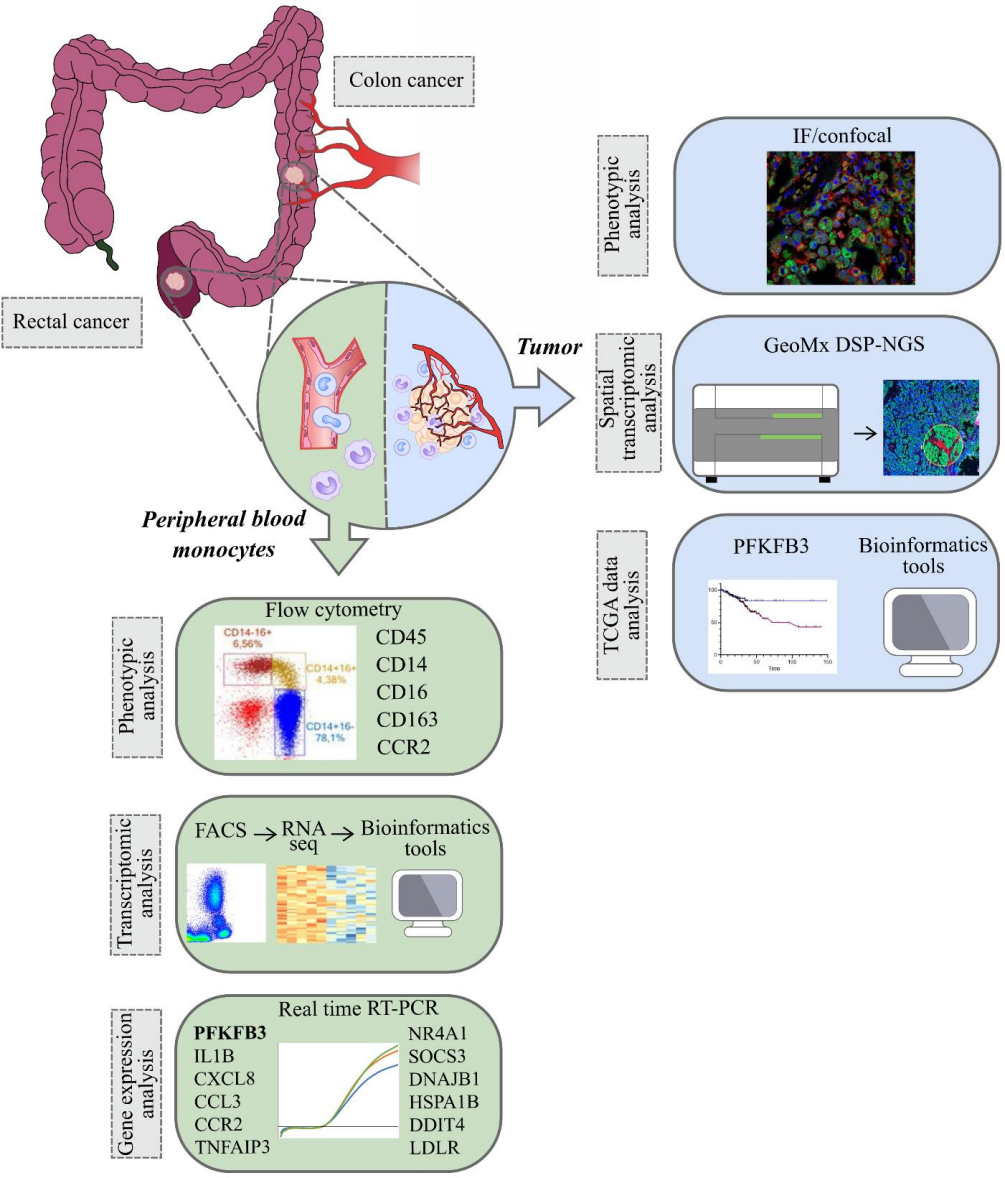
The overall study design. The samples (monocytes and tumor tissue) were collected from patients with colon and rectal cancers (1). Peripheral blood monocytes were characterized by phenotypic analysis using flow cytometry, by transcriptomic analysis using RNA sequencing (Illumina technology) and by gene expression analysis using real-time RT-PCR (2). In tumors of colon and rectum, phenotypic analysis was performed with IF/confocal microscopy. Spatial transcriptomic analysis was applied using GeoMX DSP-NGS (Nanostring technology). TCGA data were obtained for survival analysis and correlations with the parameters of progression in colon and rectal cancers. All patients` cohorts and technologies are described in the methods.

In the present study, for the first time we used RNA sequencing to identify tumor-specific programming of monocytes in CRC versus healthy individuals. We considered CRC as two clinically distinct cancers – colon cancer and rectal cancer, and, for the first time, we demonstrated different monocyte subpopulation content in patients with these two distinct intestine tumor localizations. We applied most advanced Nanostring technology to identify spatial distribution of monocyte-derived TAMs with elevated expression of the metabolic regulator. We analyzed whether monocytes can be predictive biomarkers for neoadjuvant chemotherapy (NAC) response and can have prognostic value for metastasis. We evaluated our data in the context of prognostic and therapeutic significance of monocyte programming by the design of anti-cancer immunotherapy and planning clinical trials (**Figure 1**).

## 2 Materials and methods

### 2.1 Patients

The study included patients with colorectal adenocarcinoma with morphologically verified diagnosis, treated in the Department of abdominal oncology, Cancer Research Institute of Tomsk National Research Medical Center (Tomsk, Russia) from 2019 to 2021, and healthy donors. The study was carried out according to Declaration of Helsinki (from 1964, revised in 1975 and 1983) and was approved by the local committee of Medical Ethics of Tomsk Cancer Research Institute (15 May 2019, the approval No. 6/1); all patients signed informed consent for the study. Patients were divided into two groups according to morphological diagnosis and treatment strategy: 1) patients with colon cancer who did not receive neoadjuvant chemotherapy (NAC) (T_2-4_N_0-3_M_0_, stages I–III), and 2) patients with rectal cancer underwent NAC (T_2-4_N_0-3_M_0_, stages II–III). Colon cancer included cancers of different sections of colon: the cecum, the ascending colon, the transverse colon, the descending colon, and the sigmoid colon. Rectal cancer combined localization of tumors in rectum and rectosigmoid junction. Patients with rectal cancer received 3 courses of neoadjuvant chemotherapy (NAC). Chemotherapeutic regimens included XELOX (Capecitabine plus Oxaliplatin) or FOLFOX-4 (oxaliplatin, L-leucovorin and fluorouracil). Five-grade Mandard Tumor Regression Grading (TRG) system was used for assessment of NAC response in rectal cancer patients, where TRG1 – no residual cancer, TRG2 – rare residual cancer cells, TRG3 – fibrosis outgrowing residual cancer, TRG4 – residual cancer outgrowing fibrosis, TRG5 – absence of regressive changes (21). All patients underwent surgical treatment. In adjuvant regime, patients received chemotherapy by the same schemes up to 6 months.

Healthy male and female volunteers were enrolled in this study as a control group. The age and sex of donors corresponded to patients` ones. Inclusion criteria for the healthy cohort were as follows: (a) age from 48 to 70 years, (b) no acute inflammatory diseases and severe chronic diseases (diabetes, hepatitis, HIV, myocarditis), (c) no taking immunomodulatory medication within 30 days of study (d) to be able to provide informed consent, (e) no current or past history of any oncological disease.

The material for the study was peripheral blood monocytes. For flow cytometry analysis 70 patients (34 with colon cancer and 36 with rectal cancer) and 40 healthy volunteers were enrolled. The mean age of patients with colon cancer was 66,3 ± 7,9 years, and patients with rectal cancer – 63,7 ± 8,4 years. RNA sequencing study included patients with colon (n=17) and rectal (n=12) cancers, and healthy individuals (n=19). Real time PCR analysis enrolled independent cohort of 12 patients with colon and 12 patients with rectal cancer, and 16 healthy individuals. The mean age of patients with colon cancer was 65,8 ± 11,6 years, and rectal cancer – 59,2 ± 12,1 years.

### 2.2 Monocyte isolation

Monocyte isolation was performed with FACS and CD14+ magnetic separation. For RNA sequencing, the peripheral blood mononuclear cells (PBMCs) were separated from whole blood by density gradient centrifugation using Lymphosep, Lymphocyte Separation Media (#L0560-500, Biowest, France), density 1.077 g/ml. After that, monocytes from PBMC fraction were obtained by FACS. Cells were resuspended in 150 μl of staining buffer (#2701005, Cell Staining Buffer, Sony, Japan). Monocytes were defined as CD45+CD56-CD14+7-AAD- population. Conjugated monoclonal antibodies to CD45, CD56, CD14, 7-AAD were added to the cell suspension. Samples were analyzed on a MoFlo XDP cell sorter (RRID : SCR_019665, Beckman Coulter, USA). Sorting of monocytes was carried out in the Purify 1-2 mode, the sorting efficiency was 70%, the purity of the target population was 96-99%.

Monocytes for real-time PCR were isolated from peripheral blood by density gradients followed by positive magnetic selection using CD14+ MACS beads (#130-050-201, Miltenyi Biotech, Germany), resulting to 90–98% monocyte purity as confirmed by flow cytometry.

Monocytes were obtained from the following experimental points: primary before treatment (1), after NAC (2), and after surgery (3) for patients with rectal cancer; and before surgery (1) and after surgery (2) for patients with colon cancer.

### 2.3 Flow cytometry

Whole blood samples were obtained from the healthy volunteers and for cancer patients before any treatment procedures and after four cycles of NAC (for rectal cancer only), and on 5-7 days after surgery (for colon and rectal cancer). The PBMCs were separated from whole blood by density gradient. The PBMC aliquots were incubated with optimized volumes of fluorochrome-conjugated monoclonal antibodies: CD45-APC-Cy7, CD14-Pacific Orange, CD16-APC, CD163-PE, and CCR2-PerCP-Cy5.5, with isotype controls and 7‐aminoactinomycin D (7-AAD) (BD Biosciences) to exclude the dead cells. Cells were incubated with the optimized monoclonal antibody cocktails for 15 minutes in the dark at room temperature, and then, samples were lysed using VersaLyse (#A09777, Beckman Coulter, USA). After red blood cells lysing, cells were analyzed within 20 min. For each sample, a minimum of 200.000 events were collected. Compensation procedure was performed using VersaComp antibody capture beads (#B22804, Beckman Coulter, USA). The threshold for positive staining was determined using unstained or fluorescence minus one (FMO) controls. Sample acquisition was performed on a NovoCyte flow cytometer (RRID : SCR_019522, ACEA Biosciences, USA). Data analyses were performed with NovoExpress software (ACEA Biosciences, USA).

### 2.4 RNA extraction

Total RNA was extracted from the lysed FACS-purified samples using RNeasy Plus Micro Kit (#74034, Qiagen, USA). The quality of RNA was assessed by TapeStation 4150 automated electrophoresis system (RRID : SCR_019393, Agilent Technology, USA). RNA integrity index (RIN) was 9.0-9.9. The quantity of RNA was assessed by Qubit 4 fluorometer (#RRID : SCR_018095, ThermoFisher Scientific, USA). The amount of obtained RNA was 0.4-2.8 ng/μl.

### 2.5 Whole-transcriptome RNA-sequencing

RNA libraries were prepared with NEXT flex Rapid Directional qRNA-SeqKit using indexed barcodes NEXTflex-qRNA-8nt-Barcodes (#NOVA-5198-02, Bioo Scientific, PerkinElmer Applied Genomics, USA) according manufacture`s protocols. Ribosomal RNA depletion was performed with NEBNext^®^ rRNA Depletion Kit (Human/Mouse/Rat) (NEB #E7400, New England Biolabs Inc., USA).

Whole-transcriptome sequencing was performed on total 62 samples of monocytes isolated from CRC patients. Prepared libraries were then pooled and sequenced on Illumina NextSeq500 instrument (RRID : SCR_014983, Illumina, USA) with NextSeq 500/550 High-Output v2.5 Kit (75 cycles) (#20024906, Illumina, USA). Raw data quality control was performed using FastQC (FastQC, RRID : SCR_014583) and visualized by MultiQC (MultiQC, RRID : SCR_014982) (22). Read alignment was performed using STAR aligner (STAR, RRID : SCR_004463) with GRCh38 genome and Gencode annotations (23). The numbers of reads assigned to genomic features were calculated using QoRTs software (QoRTs, RRID : SCR_018665) (24). Subsequent analysis steps were performed using DESeq2 software (DESeq2, RRID : SCR_015687) (25). Differential expression data was visualized with pheatmap (pheatmap, RRID : SCR_016418), EnhancedVolcano (EnhancedVolcano, RRID : SCR_018931), ggplot2 (ggplot2, RRID : SCR_014601), and Phantasus software (https://genome.ifmo.ru/phantasus). Fgsea (fgsea, RRID : SCR_020938) (https://www.biorxiv.org/content/early/2016/06/20/060012) and clusterProfiler (clusterProfiler, RRID : SCR_016884) (26) were used for gene set enrichment analysis of biochemical and regulatory pathways using gene lists ranked by expression level and p-value. GSEA results were visualized using ggpubr (ggpubr, RRID : SCR_021139) and GOplot (27).

Quality control revealed poor quality samples in rectal cancer group (n=1), rectal cancer after NAC group (n=8), and healthy control group (n=6). Poor quality samples were identified as outliers and were excluded from the analysis.

### 2.6 Quantitative real-time PCR

The gene expression was measured by quantitative real-time PCR using the Taqman technology and was normalized to the expression of housekeeping gene glyceraldehyde 3-phosphate dehydrogenase (GAPDH). Primers were designed using Vector NTI Advance 11.5.4 (Vector NTI, RRID : SCR_014265) program and NCBI base. Primer synthesis was carried out by the DNA-synthesis company (Moscow, Russia). The complete sequences of used primers are listed in online **Supplementary Table S1**. qRT-PCR was performed using AriaMx Real-Time PCR thermocycler (RRID : SCR_019469, Agilent Technologies, USA).

### 2.7 Immunofluorescence and confocal microscopy

Formalin fixed paraffin embedded (FFPE) tissue sections were obtained from 10 colon cancer patients. The antigen unmasking was performed using the PT Link module (Dako, Denmark) in T/E buffer (pH 9.0). For immunofluorescence (IF) staining, tumor FFPE clinical samples were treated with xylol solution and blocked with 3% BSA in PBS for 45 min, incubated with a combination of primary antibodies for 1,5 h; washed, and incubated with a combination of appropriate secondary antibodies for 45 min. Anti-PFKFB3 rabbit monоclonal antibody (1:50, #ab181861, Abcam, USA); anti-CD68 monoclonal mouse antibody (1:100, #NBP2-44539, clone KP1, Novus Biologicals); anti-CD14 polyclonal sheep antibody (1:50, #BAF383, R&D Systems) were used. Combination of secondary antibodies were applied: donkey Cy3-conjugated anti-rabbit antibody (#711-165-152, Dianova, Germany, dilution 1:400), donkey AlexaFluor488-conjugated anti-mouse antibody (#715-545-150, Dianova, Germany, dilution 1:400) and donkey AlexaFluor647-conjugated anti-sheep antibody (#A-21448, Thermo Fisher Scientific, USA, dilution 1:500). Samples were mounted with Fluoroshield Mounting Medium with DAPI (#ab104135, Abcam, USA) and analyzed by confocal microscopy. Confocal laser scanning microscopy was performed with Carl Zeiss LSM 780 NLO laser scanning spectral confocal microscope (Carl Zeiss, Germany), equipped with 40x objective. Data were acquired and analyzed with Black Zen software (RRID : SCR_018163). All four-color images were acquired using a sequential scan mode.

### 2.8 NGS-GeoMx digital spatial profiler analysis

NanoString GeoMx digital spatial profiling (DSP) was applied to perform spatially resolved RNA profiling analysis in colorectal cancer tissue. FFPE samples (5 µm) were taken from five untreated patients with colon cancer and five NAC-treated patients with rectal cancer. We used the Cancer Transcriptome Atlas (CTA) panel designed for comprehensive profiling of tumor biology, the tumor microenvironment, and the immune response. Briefly, once the slides were deparaffinised and subjected to antigen retrieval procedures, samples were co-incubated with fluorescent-labeled visualization antibodies to detect tumor cells (pan-cytokeratin [CK]), all immune cells (CD45), together with DAPI for nuclei detection. Customizable fluorescent morphology markers, conjugated to unique oligonucleotide tags with an ultraviolet (UV) photocleavable linker, helped visualizing the tissue architecture. The samples were imaged and regions of interest (ROIs) were exposed to UV light that cleaved the linker and released the barcoded oligos for capture by microfluidics and GeoMx DSP then were coupled to next generation sequencing (NGS) readout to profile RNA expression for identifying over 1,800 genes in colon and rectal cancers.

The 97 areas of illumination (AOIs) across all slides in mixed stroma/tumor regions was selected. After UV illumination, the barcodes were collected in 96-well plates and were used in the NGS-readout library preparation procedure. The resulted libraries were sequenced by the Illumina NextSeq 500 platform using 2 x 27 base paired reads. The total number of the raw reads and the average per AOI were 740M and 5M, respectively. The raw counts were processed in the NanoString’s GeoMx NGS pipeline v.2.1 where they were converted to the digital count conversion (DCC) files. The GeomxTools was used for quality control (QC) and downstream analysis of the DCC files in R. The failed QC AOIs were excluded from analysis due to sequencing quality and low number of expressed genes. The Grubbs test was used for local and global outlier probes identification. Eventually, 6 AOIs, 353 global outlier probes, and 811 local outlier probes were excluded from analysis. Genes were retrieved by probe aggregation. The low expressed gene targets were excluded regarding to the limit of quantification (LOQ). The LOQ was defined as the negative probe geomean multiplied by the 0.5 squared geometric standard deviations of the negative probes. After LOQ filtering 1367 gene targets out of 1812 were retained for further analysis. The Q3 normalization was then applied on the filtered AOIs and the gene targets. The SpatialDecon R package was used for estimating mixed cell type abundance in the AOIs. The obtained cell abundance scores were used for the Spearman’s correlation test with the Q3 normalized gene expression. The adjusted p values were calculated using the Benjamini-Hochberg correction. Difference in monocyte and macrophage cell abundance scores were tested *via* the non-parametric Mann-Whitney U test.

### 2.9 TCGA database analysis

The Cancer Genome Atlas (TCGA) data were used to examine the expression analysis and survival analysis of colorectal cancer for PFKFB3. PFKFB3 expression was evaluated in the following groups of patients (stage I-III): a) with colorectal cancer (common group) (N=417), b) with colon cancer, including transverse colon, ascending colon, descending colon, sigmoid colon, cecum, hepatic flexure, splenic flexure (n=305), c) with rectal cancer, including rectosigmoid junction and rectum (N=112), with available clinical information and records on recurrence and survival rates (in details in **Supplementary Table S2**). The TCGAbiolinks was used for retrieving RNA-seq data from the GDC database. The raw sequencing reads were processed *via* the DESeq2 R package. The raw counts were depth normalized and variance stabilized *via* the variance stabilizing transformation (VST) for downstream survival analysis. Survival analysis was performed by the ROC analysis and the Kaplan–Meier estimator in the Graph Prism 8. The difference in PFKFB3 expression between two independent groups was tested by the non-parametric Mann-Whitney U test.

### 2.10 Statistical analysis

Statistical analysis was performed using STATISTICA 8.0 for Windows (STATISTICA, RRID : SCR_014213) and GraphPad Prism 8.4.2 (GraphPad Prism, RRID : SCR_002798). The Manna-Whitney test and t-test for independent groups were implemented. The prognostic values of PFKFB3 (area under the ROC curve, confidence interval (CI), sensitivity, specificity, and threshold criteria) were determined using ROC analysis. The OS and DFS rates were determined by the Kaplan–Meier method, and the log-rank test was used to identify if the result was statistically significant. Results of real-time PCR, flow cytometry analysis and TCGA data were presented using GraphPad Prism 8.4.2 software. Results were considered to be significant with ***p<0,001, ** p<0,01 and * p<0,05. Data with marginal significance (p value <0.1) were also discussed.

## 3 Results

### 3.1 Phenotypic characterization of peripheral blood monocytes in CRC

First, we examined the differences in peripheral blood monocyte major subpopulations between CRC (CC plus RC) patients and healthy donors. Flow cytometry analysis was applied to identify tumor-associated changes in monocyte subsets in patients with CRC (N=70) compared to healthy volunteers (N=42). After that, patients with colon (N=42) and rectal (N=28) cancers were separately studied to reveal tumor localization-specific features in monocyte state. Clinical and pathological parameters of patients are summarized in **Supplementary Table S3**.

By comparison of all CRC patients and healthy donors, matched by age and gender, no statistically significant differences were identified between the percentage of major monocyte subpopulations: classical (CD14+CD16-), intermediate (CD14+CD16+) and non-classical (CD14-CD16+) (**Table 1**). By comparison of major monocyte subsets in patients with colon and rectal cancers, statistically significant decrease in the percentage of CD14-16+ monocytes was found in patients with colon cancer compared to rectal cancer (5,94 (3,38–7,6) vs. 3,62 (1,72–7,28), p=0,04) (**Table 1**).

**Table 1:** Monocyte subpopulations` content in healthy individuals and CRC patients.

Subpopulations	Healthy donors (N=42)	Cancer patients		
		Colorectal cancer (N=70)	Colon cancer (N=42)	Rectal cancer (N=28)
CD14+CD16-, %	82,06 (71,36-85,13)	83,49 (77,21-88,25)	84,89 (77,33-88,43)	82,66 (76,36-86,35)
CD14+CD16+, %	2,35 (1,89-4,16)	2,74 (2,3-7,3)	2,79 (1,8-4,12)	2,69 (1,93-4,1)
CD14-CD16+,%	5,99 (2,75-8,02)	4,62 (1,98-7,33)	3,62 (1,72-7,28) **p=0,04**	5,94 (3,38-7,6)
CD14+CD16-CD163+,%	95,27 (85,44-98,73)	89,79 (73,93-99,2)	94,44 (73,93-99,37)	87,53 (77,5-97,36)
CD14+CD16-CCR2+,%	61,00 (45,58-79,53)	70,12 (29,58-91,4)	68,47 (40,93-80,65)	85,94 (19,09-95,11)
CD14+CD16+CD163+,%	94,51 (86,89-97,87)	92,81 (81,9-96,45)	92,8 (83,89-96,45)	93,13 (80,81-96,18)
CD14+CD16+CCR2+,%	18,04 (11,88-41,84)	18,95 (10,77-48,47)	23,48 (6,55-51,85)	10,17 (2,96-18,55)
CD14-CD16+163+,%	42,12 (20,43-85,77)	56,73 (23,51-58,19)	58,88 (26,88-94,3)	43,15 (21,14-65,17)
CD14-CD16+CCR2+,%	4,39 (0,65-7,17)	5,85 (0,69-6,83)	7,25 (0,97-12,43)	2,96 (0,47-12,69)

p – difference between colon and rectal cancer groups.

We analyzed the expression of CCR2, a key chemotactic receptor on monocytes responsible for their recruitment into tumor mass (28), and CD163, a clearance receptor for hemoglobin-haptoglobin complex, which expression on TAMs correlates with tumor growth and metastasis in various cancers including CRC (6). The total amount of CD163+ and CCR2+ monocytes in all three populations was similar in healthy donors and CRC patients (**Table 1**).

Next, we analyzed the distribution of CD163 and CCR2 between three monocyte subsets. CCR2 was expressed on more than 60% of monocytes of classical CD14+CD16- subset, and the minimal expression level of CCR2 was observed in non-classical CD14-CD16+ subset in both healthy donors and cancer patients (**Table 1**). The main differences in CCR2 expression were identified by comparison of monocytes from patients with RC compared to monocytes of healthy individuals. The amount of CD14+CD16-CCR2+ monocytes in RC patients was 1,4 times higher compared to healthy individuals (85,94 (19,09–95,11) vs. 61,00(45,58-79,53)), however this difference didn`t reach statistical significance (**Table 1**). The amount of CCR2-expressing minor subsets, CD14+CD16+CCR2+ and CD14-CD16+CCR2+, were decreased (1,77 times and 1,48 times, respectively) in RC patients compared to healthy individuals. The classical subpopulation of CCR2-expressing monocytes (CD14+CD16-CCR2+) was increased in patients with RC compared to the patients with CC. Minor CCR2 expressing subsets CD14+CD16+CCR2+ and CD14-CD16+CCR2+ were decreased in patients with RC compared to patients with CC. Comparison of monocytes from patients with colon and rectal cancer showed decrease in CD14-CD16+CCR2+ in rectal cancer (fold change 2,4), however statistical significance has not been reached (**Table 1**). By comparison of monocytes of patients with CC and healthy individuals, the major difference identified was an increase of CD14-CD16+163+ monocytes in CC patients (fold change 1,4 times; 58,88(26,88-94,3) vs. 42,12(20,43-85,77)). However, this difference also didn`t reach statistical significance.

**Figure 2:**
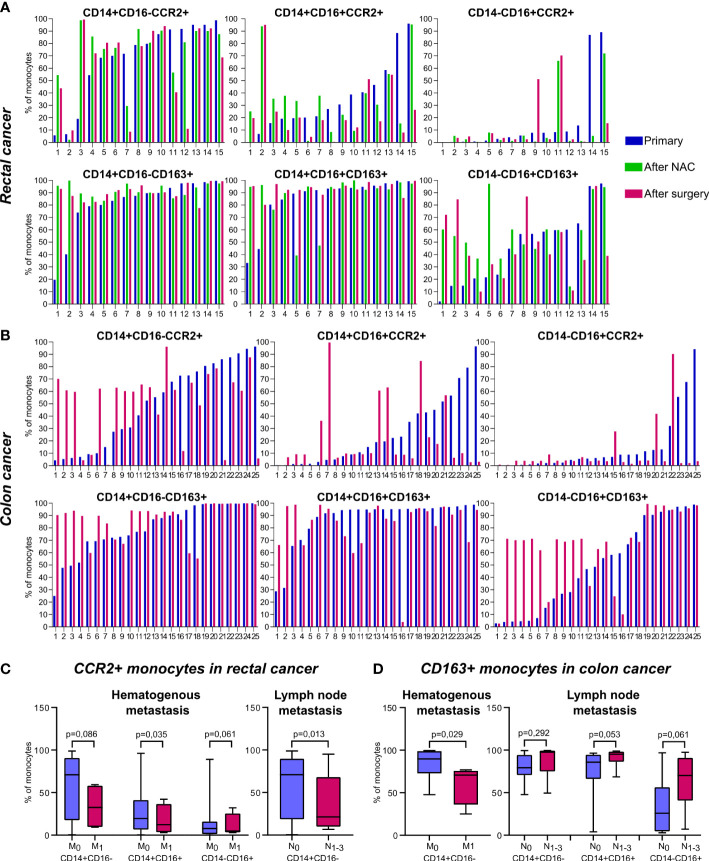
The distribution of CD163+ and CCR2+ peripheral blood monocytes in patients with colon and rectal cancers. Individual profiles of CCR2+ and CD163+ monocyte subsets for each patient with rectal and colon cancers. **(A)**, The distribution of monocytes of classical (CD14+CD16-), intermediate (CD14+CD16+) and non-classical (CD14-CD16+) populations expressing CCR2 (upper panel) and CD163 (lower panel) is demonstrated before and after NAC and after surgical resection in rectal cancer patients. **(B)**, The distribution of monocytes of classical (CD14+CD16-), intermediate (CD14+CD16+) and non-classical (CD14-CD16+) populations expressing CCR2 (upper panel) and CD163 (lower panel) is demonstrated before and after surgical resection in colon cancer patients. **(C)**, Associations of CCR2-expressing monocyte subsets with hematogenous and lymphatic metastasis in rectal cancer patients. **(D)**, Associations of CD163-expressing monocyte subsets with hematogenous and lymphatic metastasis in colon cancer patients. M_0_, metastasis-negative status, M_1_, metastasis-positive status. N_0_, lymph node-negative status, N_1-3_, lymph node-positive status.

The redistribution of CCR2+ and CD163+ monocytes between non-classical subpopulations was found in patients after treatment onset: NAC (for rectal cancer patients) and surgical resection (for rectal and colon cancer patients). Despite of overall statistical significance was not reached, to clearly identify the effect of NAC and surgery on monocyte subsets we created individual profiles for each patient (**Figure 2**).

Monocytes of classical CD14+CD16- subset didn`t show pronounced changes after NAC or surgery. In patients with rectal cancer, who had the percentage of CD14+CD16+CCR2+ monocytes below 20% out of CD14+CD16+, the percentage of CD14+CD16+CCR2+ was increased after NAC (in 6 out of 7 patients tested). In patients who had percentage of CD14+CD16+CCR2+ monocytes over 20%, the proportion of this subset was not increased after NAC (**Figure 2A**). After NAC, these patients underwent tumor surgical resection. The percentage of CD14+CD16+CCR2+ was no further changed or only slightly decreased after surgery compared to the level after NAC. For minor subpopulation CD14-CD16+ the decrease in CCR2+expression was found in patients who had high (cut-off>80%) baseline level of CD14-CD16+CCR2+ monocytes (**Figure 2A**).

In colon cancer patients, which didn’t receive NAC, and therapy consisted only out of surgical tumor resection, more pronounced difference between baseline and post-surgery levels was identified. In 9 out of 12 patients with cut-off<60% of CD14+CD16-CCR2+ cells we found the elevation of the amount of these monocyte subset after surgery, in some cases more than 6 times (**Figure 2B**). However, in patients with pre-surgery level over 60%, the percentage of CD14+CD16-CCR2+ monocytes was decreased, that was critical in two cases (19 and 17 times). The same tendency was found for CD14+CD16+ and CD14-CD16+ monocytes expressing CCR2. In case of minor CD14-CD16+ subtype, critical decrease in 23, 28 and 34 times was observed in patients with more than 50% of pre-surgery CD14-16+CCR2+ monocytes.

The subsets of CD163+ monocytes were indicative for chemotherapy impact in rectal cancer patients. Statistically significant increase in CD163+ monocytes was found in RC patients after NAC for CD14-CD16+CD163+ subset (59,9(44,46-94,51) vs. 31,79(14,88-59,8), p=0,04) and CD14+CD16-CD163+ subset (94,07(88,15-98,62) vs. 86,56(73,93-96,74), p=0,02) (data not shown). When the amount of CD14-CD16+CD163+ monocytes was below 50%, NAC stimulated the increase of this subpopulation in 100% cases. In patient 1 this elevation reached 30 times (**Figure 2A**). In colon cancer patients, high percentage of CD14+CD16- and CD14-CD16+ monocytes expressing CD163 was not changed after surgery. However, 10 out of 11 patients with colon cancer, with baseline level of CD14-CD16+CD163+ cells below 40% the percentage of this subpopulation was increased up to 18 times (**Figure 2B**).

In summary, we have identified biological valid differences in the distribution of CCR2 and CD163 expression among colon and rectal cancer patients. The major finding is both NAC and surgical intervention affect proportion of CCR2+ and CD163+ monocytes within non-classical CD14-CD16+ and CD14+CD16+ subsets in patients with CRC, and the effects are specific for colon and rectal cancer patients.

### 3.2 CCR2+ and CD163+ monocytes are specific indicators for hematogenous and lymphatic metastatic status and NAC response in patients with colon and rectal cancer

We compared all monocyte subsets before any treatment in patients who had hematogenous (TNM_1_ stage) and lymphatic (TN_1-3_M) metastasis with patients without metastasis (TNM_0_ and TN_0_M, correspondingly). The increase in total monocyte percentage was detected in lymph node (LN)-positive patients with rectal cancer (18,57 (15,86-21,74) vs. 9,43 (5,90-12,02), p=0,01). In rectal cancer, the percentage of CD14+CD16- monocytes was increased in patients with M-positive status, compared to M-negative status (85,66 (70,18-93,7) vs. 81,53 (74,59-86,07), p=0,019), while percentage of CD14-CD16+ monocytes was decreased in M-positive status, compared to M-negative status (5,98 (3,19-8,08) vs. 6,92 (1,73-13,14), p=0,07) (data not shown). The decrease in the amount of CCR2+ monocytes in all three subsets was found in RC patients with hematogenous metastasis compared to M-negative patients: 32,59 (10,08-56,81) vs. 70,90 (18,33-90,31), p=0,086 (for CD14+CD16-CCR2+); 12,28 (3,96-30,90) vs. 19,57 (6,55-40,60), p=0,035 (for CD14+CD16+CCR2+), and 4,84 (3,58-18,80) vs. 7,80 (1,46-13,72), p=0,062 (for CD14-CD16+CCR2+) (**Figure 2C**). In RC patients with lymphatic metastasis, statistically significant decrease in the percentage of classical CD14+CD16-CCR2+ monocytes was found compared to patients without lymphatic metastasis (21,26 (10,55-63,41) vs. 70,90 (18,71-88,96), p=0,013) (**Figure 2C**). However, comparing RC patients with good response to NAC (TRG_2_) and bad response to NAC (TRG_3-5_), the percentage of CD14-CD16+CCR2+ monocytes had a tendency to be increased in bad response group (7,8 (1,13-18,08), n==20 vs. 0,62 (0,40-3,89), n=4, p=0,074).

In colon cancer patients, the number of classical CD163+ monocytes negatively correlated with hematogenous metastasis (70,63 (47,08-74,51) in M_1_ vs. 83,86 (72,79-98,86) in M_0_, p=0,029). In contrast, in CC patients with lymphatic metastasis, the amount of CD163+ monocytes (3 subsets were examined) was increased compared to patients without lymphatic metastasis: 87,59 (76,86-99,36) vs. 79,56 (70,72-94,31), p=0,29 (for CD14+CD16-CD163+), 91,48 (86,50-98,05) vs. 77,81 (66,21-94,53), p=0,053 (for CD14+16+163+), and 64,28 (46,67-90,37) vs. 34,18 (4,50-56,29), p=0,061 (for CD14-16+163+) (**Figure 2D**).

In summary, we found that in patients with rectal cancer increased amount of CCR2+ monocytes was indicative for the absence of both lymphatic and hematogenous metastasis. In contrast, in patients with colon cancer CD163+ monocyte population was indicative for LN metastasis development. CD14-CD16+CCR2+ subpopulation can be predictive for bad NAC response in rectal cancer.

### 3.3 Colorectal cancer induces transcriptional programming in human monocytes

**Figure 3:**
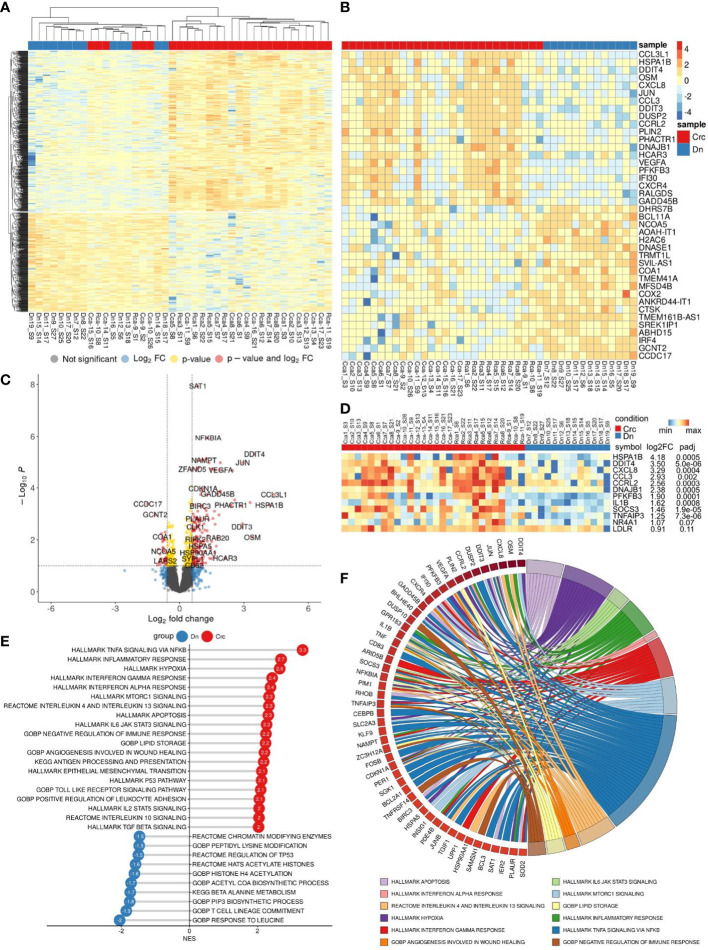
Comparative transcriptome of monocytes from CRC patients (Crc) and healthy donors (Dn). **(A)**, Heatmap demonstrates hierarchical clustering of samples and upregulated and downregulated genes in monocytes of CRC patients (FDR<0.25). **(B)**, Heatmap with top 20 DEGs upregulated and downregulated in monocytes of CRC patients (FDR<0.1). **(C)**, Volcano plot shows p-value and log2 fold-change value for DEGs in monocytes of CRC patients (|L2FC|>0.58, FDR<0.1). **(D)**, Set of genes from DEA analysis selected for RT-PCR validation. **(E)**, Bar plot with GSEA results demonstrates top deregulated pathways in monocytes of CRC patients (FDR<0.1). **(F)**, Chord plot demonstrates top upregulated pathways (FDR<0.01) and their top enriched genes (FDR<0.05) in CRC monocytes.

We performed bulk RNA sequencing (RNAseq) on total 62 samples of monocytes isolated from CRC patients. The study included patients with colon (n=17) and rectal (n=12) cancers, rectal (n=14) cancer after NAC, and healthy individuals (n=19). Differential expression analysis (DEA) was performed by comparing monocytes of CRC patients with monocytes of healthy controls (Dn) (**Figure 3A**).

Principal component analysis (PCA) and hierarchical clustering separated the transcriptome of CRC monocytes from the transcriptome of Dn monocytes. DEA revealed 232 (353) upregulated and 84 (174) downregulated genes in CRC monocytes (false discovery rate (FDR)<0.05 (0.1)). The top significant genes are demonstrated by heatmap at **Figure 3B**. Volcano plot shows genes (|Log2FC| > 0.58, FDR<0.1) expression of which was deregulated in CRC monocytes (**Figure 3C**).

Gene set enrichment analysis (GSEA) was performed for up- and downregulated genes in CRC monocytes. Pathways with normalized enrichment score |NES|>1.50 and FDR<0.1 were considered. All genes were distributed in groups according to the biochemical and functional pathways using the following databases REACTOM, KEGG, HALLMARK, GO BP. The TNFa signaling, inflammatory response and hypoxia were the top upregulated pathways, along with mTORC1 signaling, IL-4, IL-10 and IL-13 signaling, angiogenesis involved in wound healing, TGFb signaling, TLR pathways, and epithelial-mesenchymal transition (EMT) (**Figure 3E**). The downregulated genes were enriched in processes mainly associated with chromatin remodeling, DNA conformation, T-cell linage commitment pathway, and histone post-translational modification (**Figure 3E**). Top upregulated pathways (FDR<0.01) and their top enriched genes (FDR<0.05) in CRC monocytes are demonstrated in chord plot (**Figure 3F**).

Taken into account different clinical characteristics of colon and rectal cancers, we divided all patients into two groups and distinctly compared the transcriptome of monocytes of colon and rectal cancers with monocytes of healthy donors. DEA for monocytes of colon and rectal patients with monocytes of healthy individuals separately are available as supplementary materials (**Supplementary Figures S1, S2**). Overlapping pathways were identified to be affected in monocytes of patient with colon and rectal cancer compared to monocytes of healthy individuals. We next applied several bioinfomatical methods to identify possible differences in transcriptome of monocytes in patients with colon and rectal cancer.

### 3.4 Comparative bioinformatical analysis of the transcriptome of monocytes in patients with colon and rectal cancers before therapy

**Figure 4:**
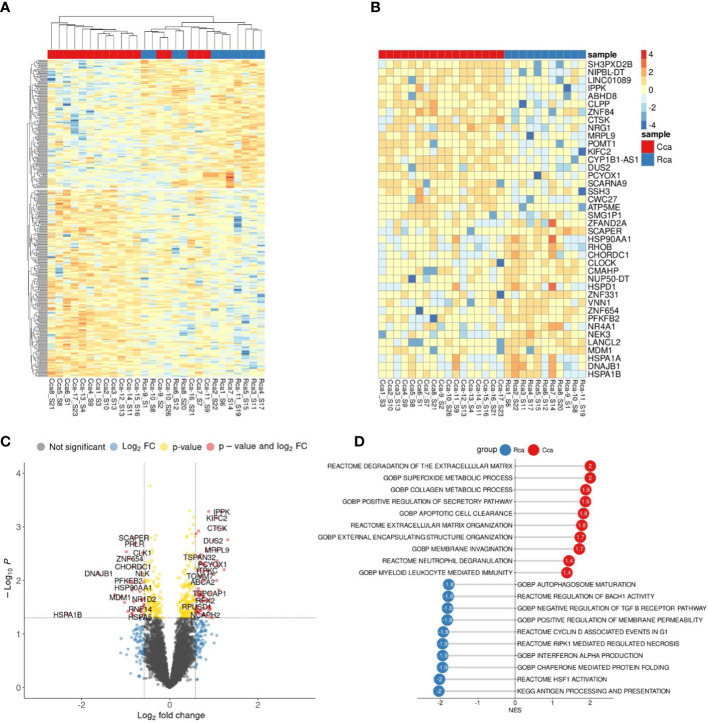
Comparative transcriptome of monocytes from colon cancer (CC) and rectal cancer (RC) patients. **(A)**, Heatmap demonstrates hierarchical clustering of samples and upregulated and downregulated genes in monocytes of CRC patients (FDR<0.25). **(B)**, Heatmap with top 20 DEGs in monocytes of colon and rectal cancers (FDR<0.25). **(C)**, Volcano plot shows p-value and log2 fold-change value for DEGs in monocytes of colon and rectal cancers (|L2FC|>0.58, FDR<0.25). **(D)**, Bar plot with GSEA results demonstrates top deregulated pathways in monocytes of colon and rectal cancers (FDR<0.25).

To evaluate the presence of differences between the transcriptome of colon cancer monocytes and rectal cancer monocytes we performed DEA (**Figure 4A**). PCA and hierarchical clustering did not noticeably distinguish the differences in CC monocytes and RC monocytes. DEA revealed no genes with FDR<0.1. Top genes with failed FDR are demonstrated by heatmap and volcano plot (**Figures 4B, C**).

Nevertheless, using GSEA we were able to find significant pathways with normalized enrichment score |NES|>1.40 and FDR<0.25, that are enriched by set of genes. The top upregulated pathways in CC monocytes were degradation of extracellular matrix (ECM), collagen metabolic process, superoxide metabolic process, apoptotic cell clearance, and membrane invagination. The top upregulated pathways in RC monocytes were antigen processing and presentation, HSF1 activation, interferon alpha production, regulation of response to interferon gamma, and synthesis of PIPs at the plasma membrane (**Figure 4D**). Although there are no statistically significant genes with FDR<0.1, GSEA identified statistically significant difference (FDR<0.25) in pathway enrichment in CC and RC monocytes. Our data suggest that monocytes in CC are programmed towards clearance and matrix remodeling activity, while monocytes in rectal cancer are programmed more towards pathogen response.

### 3.5 Chemotherapy affects monocyte profile in rectal cancer

**Figure 5:**
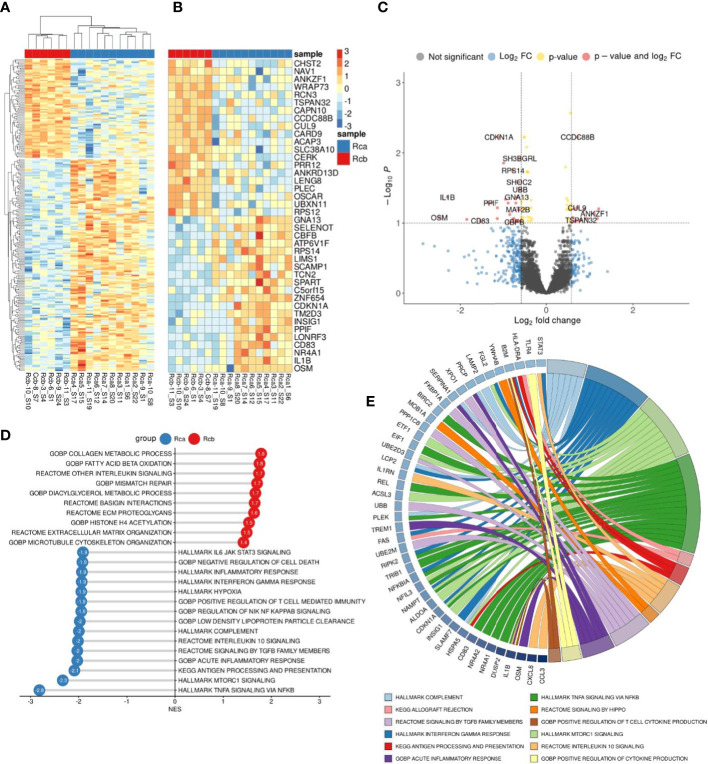
Comparative transcriptome of monocytes from rectal cancer patients before (Rca) and after (Rcb) neoadjuvant chemotherapy. **(A)**, Heatmap demonstrates hierarchical clustering of samples and upregulated and downregulated genes in RC patients after NAC (FDR<0.25). **(B)**, Heatmap with top 20 DEGs upregulated and downregulated in monocytes of in RC patients after NAC (FDR<0.1). **(C)**, Volcano plot shows p-value and log2 fold-change value for DEGs in monocytes of RC patients after NAC (|L2FC|>0.58, FDR<0.1). **(D)**, Bar plot with GSEA results demonstrates top deregulated pathways in monocytes of in RC patients after NAC (FDR<0.1). **(E)**, Chord plot demonstrates top upregulated pathways (FDR<0.01) and their top enriched genes (FDR<0.05) in RC patients after NAC.

DEA analysis was performed for the monocytes before and after NAC in rectal cancer (**Figures 5A–C**). GSEA was performed for up- and downregulated genes in monocytes of rectal cancer under NAC effect. Pathways with normalized enrichment score |NES|>1.40 and FDR<0.1 were considered. The top upregulated processes in post-NAC fraction of monocytes are collagen metabolic processes, interleukin signaling, fatty acid beta and lipid oxidation, microtubule formation, histone acetylation (**Figure 5D**). The inhibited pathways included TNFa signaling, mTORC1 signaling, inflammatory response, antigen processing and presentation, signaling by TGF-b, IL-10 signaling, hypoxia, IL-6 JAK STAT3 signaling (**Figure 5D**). Interestingly, NAC revoked monocyte programming that is induced in response to tumor growth and can be beneficial for patients, and programmed monocytes towards M2-phenotype that is typical for TAMs of other solid cancers and that supports tumor progression. Top downregulated pathways (FDR<0.01) and their top enriched genes (FDR<0.25) under NAC effect in Rcb monocytes are demonstrated in chord plot (**Figure 5E**). Key effects of NAC on monocyte transcriptome included inhibition of cytokine pathways and immune responses and stimulation of M2-associated metabolism and histone modifications.

### 3.6 Validation of RNAseq results revealed significant changes in transcriptomic profile of monocytes in patients

PCR validation of RNAseq results confirmed differential expression of following genes: *DDIT4, CCRL2, CCL3, PFKFB3, TNFAIP3, SOCS3, LDLR, DNAJB1, HES4, IL1b, CXCL8, NR4A1*, and *HSPA1B* (**Figure 3D**). These genes belong to chemokines and cytokines, heat shock proteins, metabolic regulators, transcription factors and DNA-binding proteins. The expression of these genes was assessed in similar independent cohorts: common group of patients with CRC (N=23) (**Supplementary Figure S5**), patients with rectal cancer before (N=11) and after (N=9) NAC (**Supplementary Figure S3**), patients with colon cancer before (N=12) and after (N=14) surgery (**Supplementary Figure S4**).

The significant differences were demonstrated for the baseline expression of *PFKFB3, NR4A1* and *IL1b*, which was more than 2 times upregulated in colon cancer compared to healthy control (0,52 (0,43-1,04) vs. 0,23 (0,18-0,36), p= 0,0007; 2,10 (0,83-4,30) vs. 0,30 (0,20-2,75), p=0,02, and 1,60 (0,76-2,82) vs. 0,18 (0,07-0,46), p=0,00081, relatively) (**Supplementary Figure S5**). This increase was also detected in common CRC group of patients. The expression of *IL1b* was 2,6 times increased in RC patients compared to healthy individuals (1,27 (,0,42-1,61) vs. 0,48 (0,08-0,46) (p=0,005). The expression of PFKFB3 was 2,5 times elevated in colon cancer compared to rectal cancer (0,76 (0,21-0,71) vs 0,31 (0,19-0,37) (p=0,003) (**Supplementary Figure S5**). Interestingly, post-surgery levels of *PFKFB3, IL1b, HSPA1B* and *DDIT4* in colon cancer were increased compared to control (**Supplementary Figure S4**). For rectal patients only upregulation of baseline and post-NAC levels of *IL1b* were detected compared to control (**Supplementary Figure S3**). The decrease in *CCL3* and *DNAJB1* was found after NAC (**Supplementary Figure S3**).

### 3.7 Nanostring technology identified PFKFB3+ monocytes as a key source of TAMs in colon cancer

**Figure 6:**
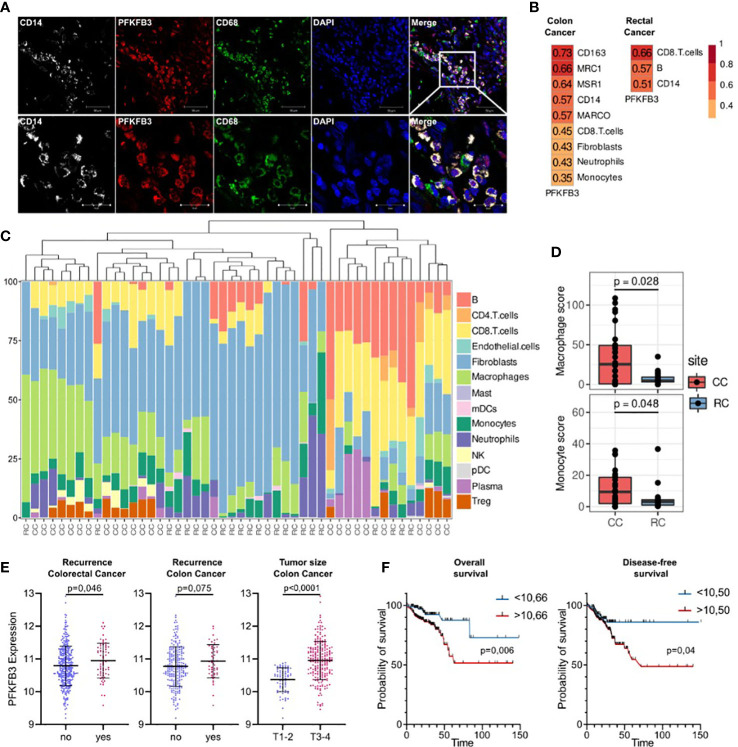
An activator of glycolysis PFKFB3 is overexpressed in colon cancer and is indicative for higher risk of tumor relapse in colon cancer but not rectal cancer. **(A)**, Сolon cancer tissue is massively infiltrated by PFKFB3-positive monocytes. IF/confocal microscopy analysis was performed for 10 colon tumor tissues. The infiltration of CD14+CD68+PFKFB3+ cells was found in all samples. Representative image is given from one patient. Scale bar corresponds to 50 µm in main image and 20 µm in zoom image. **(B)**, Spearman correlation coefficients between PFKFB3 expression, M2 macrophage gene expressions and predicted cell abundance scores, FDR<0.05. **(C)**, Predicted cell composition of CD45+ AOIs and hierarchical clustering of AOIs. **(D)**, Difference in monocyte and macrophage cell abundance scores between the CD45+ AOIs in colon and rectal cancers (the Mann-Whitney U test was applied). **(E)**, PFKFB3 gene expression is elevated in patients with recurrence and larger tumor size in colon cancer. Variance in PFKFB3 expression was stabilized *via* the variance stabilizing transformation (VST). **(F)**, PFKFB3 had prognostic significance for the DFS and OS. High-risk group had worse survival rates compared to low-risk group. ROC analysis and Kaplan–Meier method were applied.

We focused on glycolytic activator 6-phosphofructose-2-kinase and fructose-2,6-bisphosphatase (PFKFB3), which demonstrated most significant differences in colon cancer. PFKFB3 is a regulator of metabolic switch in macrophages, was found in LPS-activated M1 macrophages (29). We questioned whether PFKFB3-positive monocytes infiltrate colon cancer tissue. We performed IF/confocal microscopy analysis in tumor tissues of 10 patients with colon cancer. It was demonstrated that PFKFB3 is predominantly expressed on CD14+CD68+ monocyte-derived macrophages that massively infiltrate tumor tissue (**Figure 6A**).

Next question we asked whether monocytes is a major source of TAMs in colon and rectal cancer. We applied GeoMX DSP (Nanostring technologies) coupled with NGS readout to perform profiling of RNA expression of over 1,800 genes. GeoMX DSP allowed us to apply spatial transcriptomic analysis on FFPE tumor samples taken from NAC-treated RC patients and untreated CC patients. We found that PFKFB3 expression strongly correlated with the expression of CD14 (monocyte marker), CD163 (marker of monocytes and immature monocyte-derived macrophages) and receptor markers of M2 polarization: CD206 [MRC1], CD204 [MSR1] and MARCO (**Figure 6B**). We also found significant correlation of PFKFB3 expression with myeloid cell population, as well as with CD8 T cells, fibroblasts and neutrophils. We performed separate analysis of colon and rectal cancer samples, and found that listed above correlations are specific only for colon cancer. In rectal cancer, correlation of PFKFB3 expression with CD14, but not with M2-like markers was found (**Figure 6B**). Enrichment by macrophages and monocytes was more pronounced in colon cancer compared to rectal cancer (p=0,006 and p=0,049, correspondingly) (**Figures 6C, D**).

### 3.8 Prognostic significance of PFKFB3 expression in colon cancer but not rectal cancer

On the final step, we assessed the prognostic significance of PFKFB3 in colorectal cancer. The high PFKFB3 expression level was significantly associated with recurrence in patients with colorectal cancer (p=0,046). The same tendency was found for colon, but not rectal cancer (p=0,075) (**Figure 6E**). Moreover, PFKFB3 expression was upregulated in colon cancer patients with larger tumor size (T3-4) compared to patients with smaller tumor size (T1-2) (FC=1,4, p<0,0001) (**Figure 6E**). The association between the PFKFB3 and disease-free survival (DFS) and overall survival (OS) was determined by ROC curve analysis. A cut-off values of 10,50 for prognosis of DFS and 10,66 for OS were established. In DFS analysis, the corresponding sensitivity was 82,35%, the specificity was 31,8%, and the area under the curve (AUC) was 0.586 (95%CI: 0.514–0.657, p=0,025). Therefore, we divided the patients into two groups (a high PFKFB3 group ≥ 10,50 and low PFKFB3 group < 10,50). The DFS rate of the low PFKFB3 group was higher than that of the high PFKFB3 group (p=0,04, **Figure 6F**). In OS analysis, the corresponding sensitivity was 79,09%, the specificity was 45,04%, and the AUC was 0.616 (95%CI: 0.531–0.703). We divided the patients into two groups (a high PFKFB3 group ≥ 10,66 and low PFKFB3 group < 10,66). In low PFKFB3 group the OS rate was higher than that of the high PFKFB3 group (p=0,006, **Figure 6F**). In rectal cancer cohort, PFKFB3 was not significantly associated with outcome. Thus, PFKFB3 was found as a prognostic factor for tumor relapse and unfavorable outcome in patients with colon cancer, but not with rectal cancer.

## 4 Discussion

Monocytes are innate immune cells belonging to mononuclear phagocyte system that serve as important regulators of cancer development and progression, and can be programmed also by cancer therapy (14, 29). Human monocytes are heterogenic and can be defined by various markers, such as HLA-DR, CX3CR1, CCR2, CD62L, Tie2, CD86, CD206, and others (30–32). Using flow cytometry we investigated the distribution of CCR2 and CD163 expression on classical (CD14+16-), intermediate (CD14+16+) and non-classical (CD14+16++) monocytes` subsets. Chemokine CCL2, the main monocyte chemoattractant, determines the mobilization of monocytes from bone marrow into the blood and recruitment to the tissue from the bloodstream (14, 28). TAMs recruited in CCR2-dependent manner contribute to the tumor development (33). An accumulation of CD163+ monocytes was considered as a marker of high malignancy in CRC, however, the level of CD163 expression on monocytes was not associated with clinical outcome (34). Our own previous results on breast cancer (BC) patients revealed that the percentage of CD163-expressing CD14-CD16+ and CD14+CD16+ monocyte subpopulations was higher in BC patients compared to healthy women (19).

Here we have identified biological valid differences in the distribution of CCR2 and CD163 expression on monocytes among colon and rectal cancer patients. Both NAC and surgical intervention affected proportion of CCR2+ and CD163+ monocytes within non-classical CD14-CD16+ and CD14+CD16+ subsets in patients with CRC, and effects were specific for colon and rectal cancer patients. We demonstrated that in patients with rectal cancer increased percentage of CCR2+ monocytes was indicative for the absence of both lymphatic and hematogenous metastasis. In contrast, in patients with colon cancer, CD163+ monocyte population was indicative for higher risk of lymphatic metastasis.

These findings provide essential argument towards CRC definition to cover two clinically distinct cancers – colon cancer (including cancers of different sections of colon: the cecum, the ascending colon, the transverse colon, the descending colon, the sigmoid colon) and rectal cancer (combined localization of tumor in rectum and recto-sigmoid junction), that differentially interact with innate immunity. Convincing clinical evidences showed that these two cancers have to be considered as two different entities due to their topography, surgical challenge, therapy, complications, and relapse pattern (3).

In the present study, using RNAseq of peripheral blood monocytes we identified tumor-specific programming of monocytes in patients with CRC. We compared transcriptomic profile of CRC monocytes and monocytes of healthy donors. Genes with upregulated expression were preferentially functionally attributed to inflammatory and M2-associated signaling. There are limited data about tumor-educated monocytes in cancer patients. In breast cancer (BC), RNAseq revealed increased expression of transcripts encoding immune regulatory receptors (CD200R1), pro-apoptotic molecules (TNFSF10), and pro-angiogenic factors (HGF and ANGPT1) in BC patients compared to healthy donors (17). Using microarray analysis, blood monocytes from renal cell carcinoma (RCC) patients and healthy donors were compared (35). Compared to monocytes of healthy controls, RCC monocytes consistently displayed upregulation of pro-inflammatory cytokine and chemokine genes and genes, associated with pro-tumor polarization (35). Overlapped with our RNAseq results genes include IL1b, CCL3, CXCL8, VEGFA and CXCR4 (35). RNA microarray analysis allowed to find distinct gene signature in monocytes of CRC patients in comparison with healthy individuals (36). After validation, a prognostic panel consisting of 23 genes was established. However, this data set was not powered to address the question about clinical value of these genes with sufficient statistical significance (36). Hamm A. et al. in their study have not found the differences between monocytes of metastatic and non-metastatic CRC, but this study did not discriminate between colon and rectal cancer (36). The effect of different types of therapy was also not studied.

In our study for the first time, we have compared monocytes of colon and rectal cancer as separate types of tumor localization, and examined effect of both NAC and surgery on monocyte transcriptional profile. We identified chemotherapy-induced programming of monocytes in these patients. Cytostatic agents are able to modulate the recruitment of monocytes into the tumor, their differentiation into specific TAM populations and their participation in adaptive antitumor immune response (37, 38). Such modulation can dramatically affect tumor progression after chemotherapy, contributing to poor response and poor outcome. Our data show that monocytes of rectal cancer patients before therapy onset develop rather beneficial transcriptional program (compared to colon cancer patients) that would allow monocyte-derived TAMs to retain anti-tumor activity. However, NAC in rectal cancer patients revoked monocyte program towards M2-phenotype that is typical for TAMs of other solid cancers, and that supports tumor progression and TAM-mediated therapy resistance (6, 39, 40).

RNAseq analysis validated by RT-PCR allowed to identify specific upregulation of glycolytic activator PFKFB3 monocytes of colon cancer patients compared to healthy individuals and to patients with rectal cancer. PFKFB3 was initially identified in human macrophages in the mid-1990s as a vital regulator of glycolysis (41). Rapidly proliferating cancer cells constitutively express PFKFB3 *in vitro*, and inhibition of PFKFB3 expression decreases tumor growth in experimental animal models (42). In number of cancers, PFKFB3 expression was increased compared to normal tissue (41). The altered metabolism of cancer cells is called the Warburg effect and is characterized by an increase in glycolysis (43, 44). In macrophages, activation of the NLRP3 inflammasome and the release of IL1β, play key role in modulating glycolysis *via* PFKFB3 (45). A number of PFKFB3 inhibitors demonstrated efficacy in reducing tumor growth in several tumor models, including melanoma, lung, colon, pancreatic, gastric and breast cancers (46, 47). PFKFB3 was found to be central element of the mechanism of metabolic switch that regulates the pro-tumor programming of monocytes in human hepatocellular carcinoma (48). However, in breast and in colorectal cancer, despite large number of studies demonstrating critical role of PFKFB3 in cancer cells metabolism and proliferation, and despite the ongoing studies on the therapeutic targeting of PFKFB3, its role in the programming of immune system on systemic or local levels is largely unexplored (49, 50).

Our data for the first time demonstrate that colon cancer affects circulating monocytes transcriptome and induces elevation of PFKFB3 expression. Confocal microscopy demonstrated that PFKFB3-positive monocytes, that are precursors of TAMs, massively infiltrate tumor mass. GeoMX DSP-NGS analysis of colon cancer patients identified the correlation of PFKFB3 expression with monocytes infiltration and M2-type polarization markers, including CD163, CD206, CD204 and MARCO. Cell type analysis revealed that monocyte and macrophage cell count is more abundant in colon cancer tissue compared to rectal cancer tissue. Finally, we showed that PFKFB3 expression is a significant prognostic factor for poor OS and DFS and for high risk of tumor relapse in colon but not rectal cancer patients.

Overall, for the first time we defined the key differences in monocyte programming and their potential to give origin to tumor-supporting TAMs in colon versus rectal cancer. The essential feature of monocytes in colon cancer patients is their transcriptional program supporting activation of glycolytic pathway that correlates with the pro-tumoral phenotype of monocyte-derived TAMs and with unfavorable prognosis for patients. The differences in monocyte phenotype between colon and rectal cancers can be potentially explained by specific immune status of tumors in colon and rectal compartments. At this stage we can hypothesize that distinct immune status of colon and rectal cancer is affected by the exposure of immune cells to distinct metabolic composition of the processed food from one side, and by distinct composition and metabolism of microbiota in these two compartments of the digestive tract. Attractive is the fact that monocytes are located in the circulation, that makes them accessible as minimally invasive biomarkers and therapeutic targets (14). Recent studies using high throughput technologies have revealed that transcriptional alterations in peripheral blood monocytes can serve as diagnostic, predictive and prognostic biomarkers in renal, colorectal, breast, cervical, skin, thyroid, hepatocellular and lung cancers (35, 36, 51–56). Our data open the perspective for the differential development of monocyte/macrophage targeted immunotherapy for patients with colon and rectal cancer.

## Data availability statement

The datasets presented in this study can be found in online repositories. The names of the repository/repositories and accession number(s) can be found below: RNAseq (GSE221925): https://www.ncbi.nlm.nih.gov/geo/query/acc.cgi?acc=GSE221925; GeoMx (GSE221924): https://www.ncbi.nlm.nih.gov/geo/query/acc.cgi?acc=GSE221924.

## Ethics statement

The studies involving human participants were reviewed and approved by Medical Ethics of Tomsk Cancer Research Institute. The patients/participants provided their written informed consent to participate in this study.

## Author contributions

IL: Conceptualization, data curation, formal analysis, validation, investigation, visualization, methodology, writing-original draft, project administration. MP: Software, formal analysis, validation, investigation, visualization. PI: Resources, software, formal analysis, validation. EK: Formal analysis, validation, visualization. AnK: Validation, visualization, methodology. MR: Validation, visualization, methodology. EG: Validation, visualization. AT: Validation, methodology. SA: Validation, visualization. NB: Validation, visualization. ArK: Software, resources. AD: Validation, methodology. DK: Validation, methodology. NC: Validation, project administration. JK: Conceptualization, data curation, supervision, project administration, writing-review and editing. All authors contributed to the article and approved the submitted version.
