# Supplementary material for: PFKFB3 overexpression in monocytes of patients with colon but not rectal cancer programs pro-tumor macrophages and is indicative for higher risk of tumor relapse
Source: Front Immunol. 2023 Jan 17;13:1080501. doi: 10.3389/fimmu.2022.1080501 (PMC9887047; doi:10.3389/fimmu.2022.1080501)
Supplement: Supplementary file 1 [file DataSheet_1.docx]

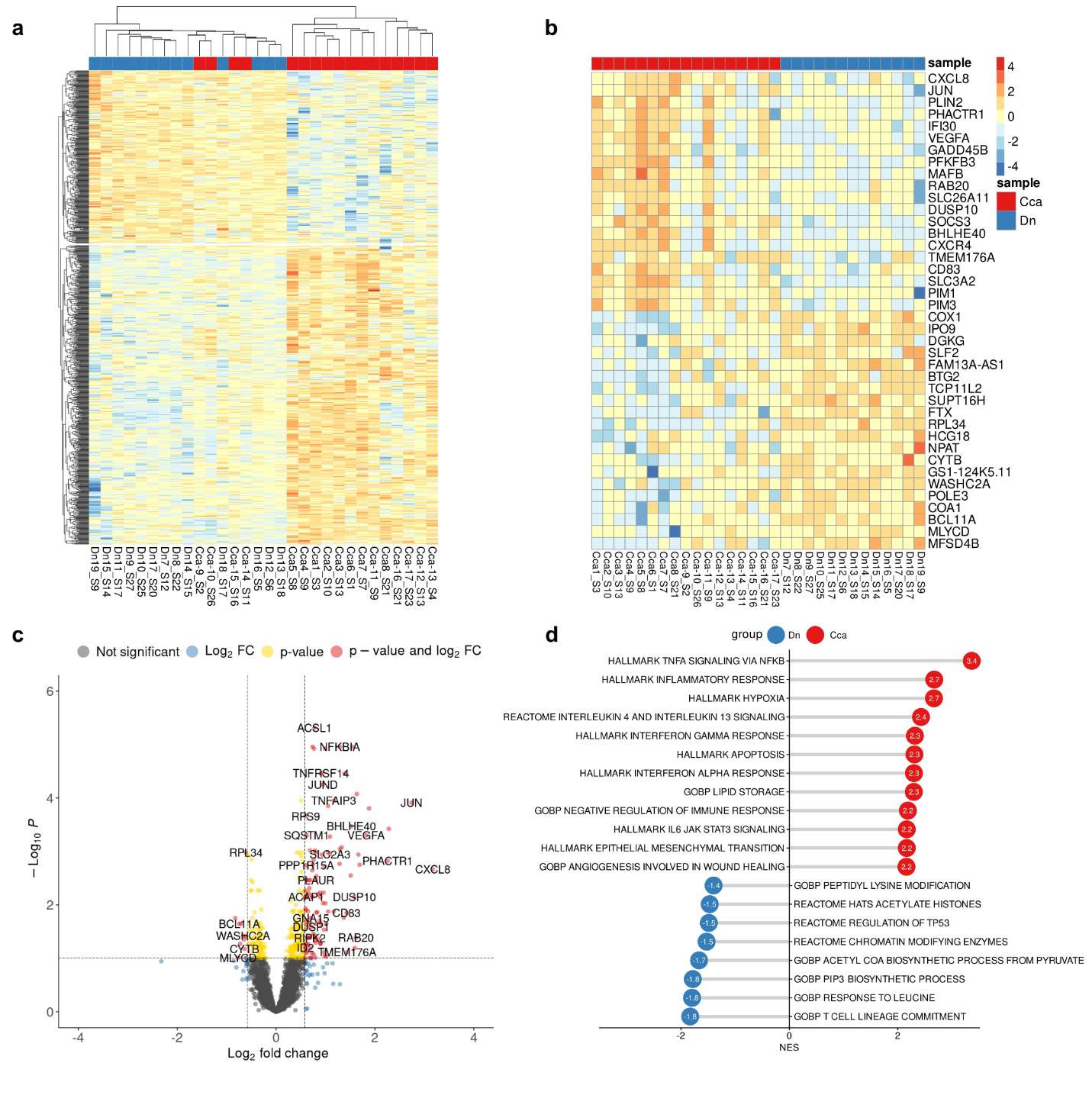


**Supplemental Figure S1. Comparative transcriptome of monocytes from colon cancer patients (Cca) and healthy donors (Dn).** **a,** Heatmap demonstrates hierarchical clustering of samples and upregulated and downregulated genes in monocytes of CC patients (FDR<0.25). **b,** Heatmap with top 20 DEGs upregulated and downregulated in monocytes of CC patients (FDR<0.1). **c,** Volcano plot shows p-value and log2 fold-change value for DEGs in monocytes of CC patients (|L2FC|>0.58, FDR<0.1). **d,** Bar plot with GSEA results demonstrates top deregulated pathways in monocytes of CC patients (FDR<0.1).


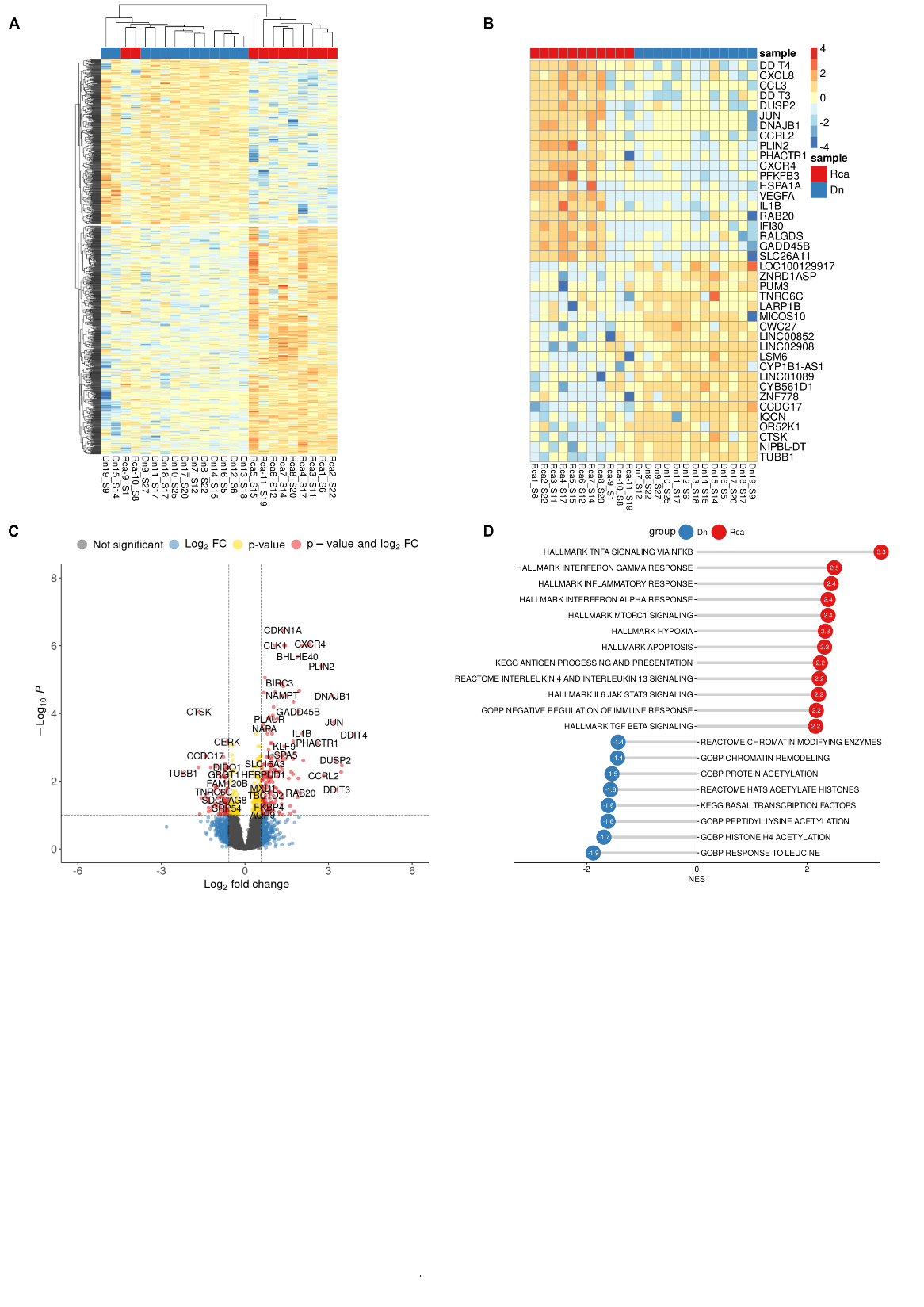


**Supplemental Figure S2. Comparative transcriptome of monocytes from rectal cancer patients (Rca) and healthy donors (Dn).** **a,** Heatmap demonstrates hierarchical clustering of samples and upregulated and downregulated genes in monocytes of RC patients (FDR<0.25). **b,** Heatmap with top 20 DEGs upregulated and downregulated in monocytes of RC patients (FDR<0.1). **c,** Volcano plot shows p-value and log2 fold-change value for DEGs in monocytes of RC patients (|L2FC|>0.58, FDR<0.1). **d,** Bar plot with GSEA results demonstrates top deregulated pathways in monocytes of RC patients (FDR<0.1).


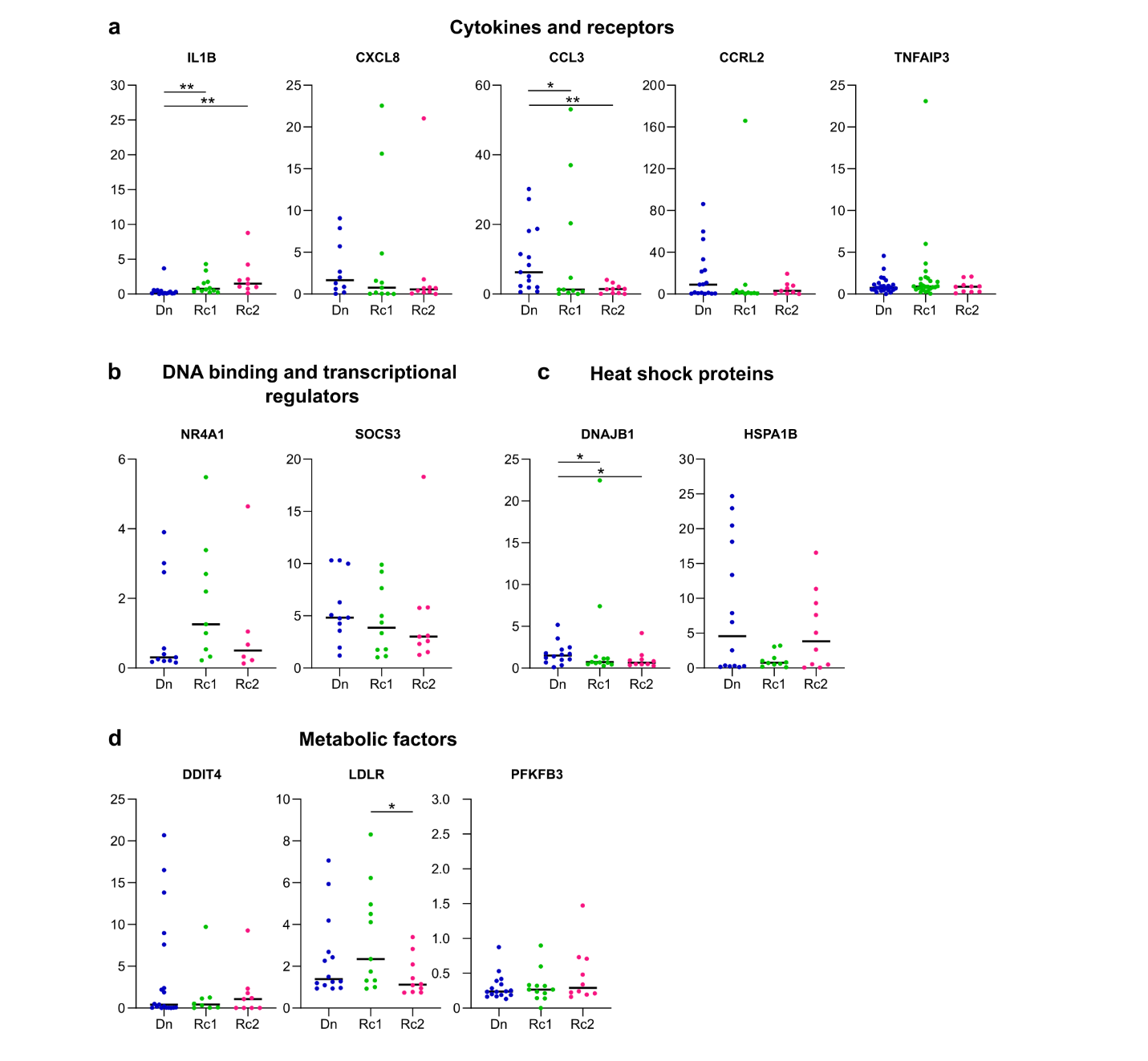


**Supplemental Figure S3. Validation of RNAseq results for rectal cancer patients before and after NAC.** Real-time PCR analysis was performed for the comparison of the expression of validation gene set in colon cancer patients before (Rc1) and after (Rc2) NAC and for healthy individuals (Dn). Genes were functionally attributed to cytokines and receptors (**a**), DNA binding and transcriptional regulators (**b**), heat shock proteins (**c**) and metabolic factors (**d**). * - p<0,05, ** - p<0,01, *** - p<0,001.


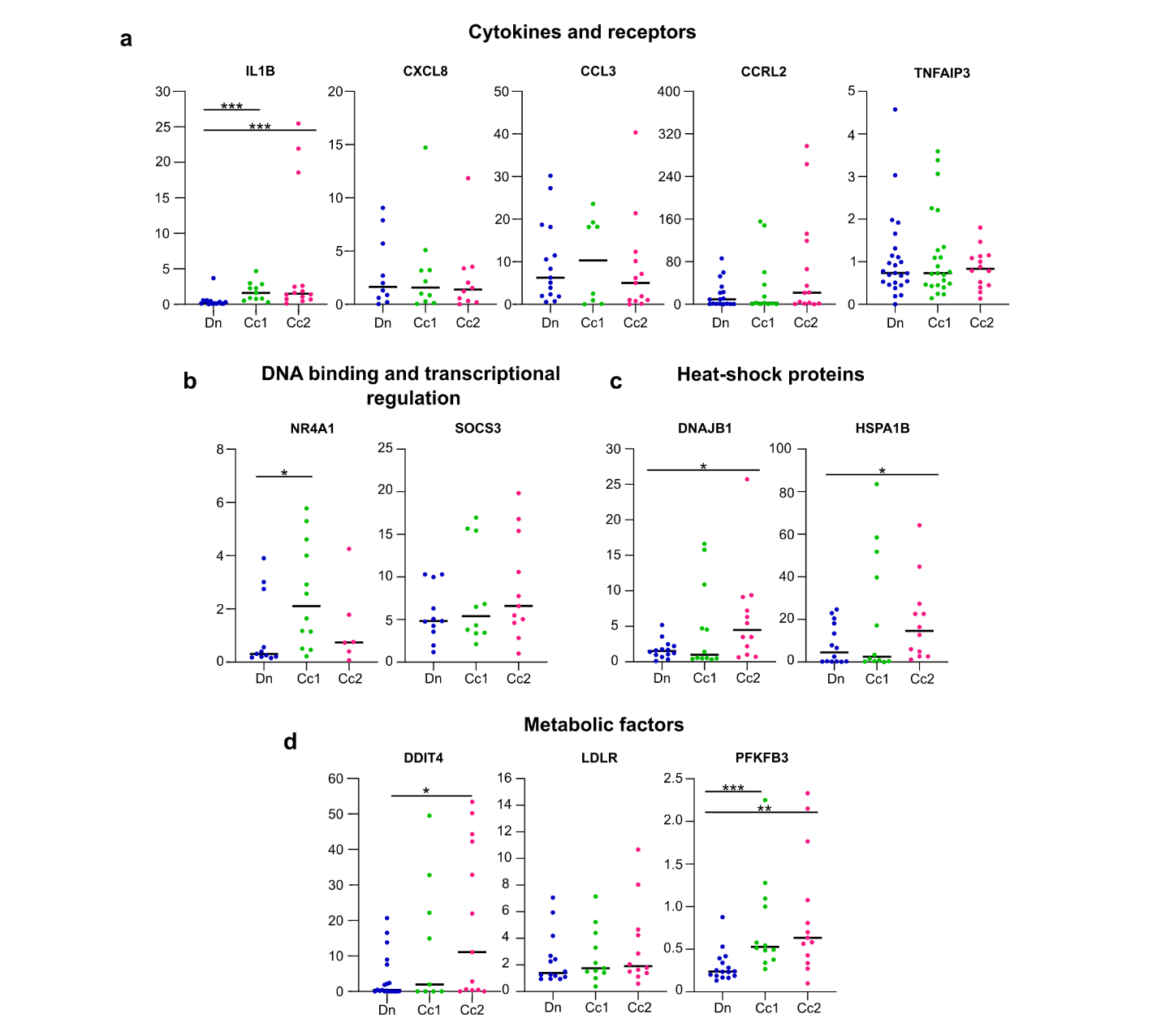


**Supplemental Figure S4. Validation of RNAseq results for colon cancer patients before and after surgical resection.** Real-time PCR analysis was performed for the comparison of the expression of validation gene set in colon cancer patients before (Cc1) and after (Cc2) tumor resection and for healthy individuals (Dn). Genes were functionally attributed to cytokines and receptors (**a**), DNA binding and transcriptional regulators (**b**), heat shock proteins (**c**) and metabolic factors (**d**). * - p<0,05, ** - p<0,01, *** - p<0,001.


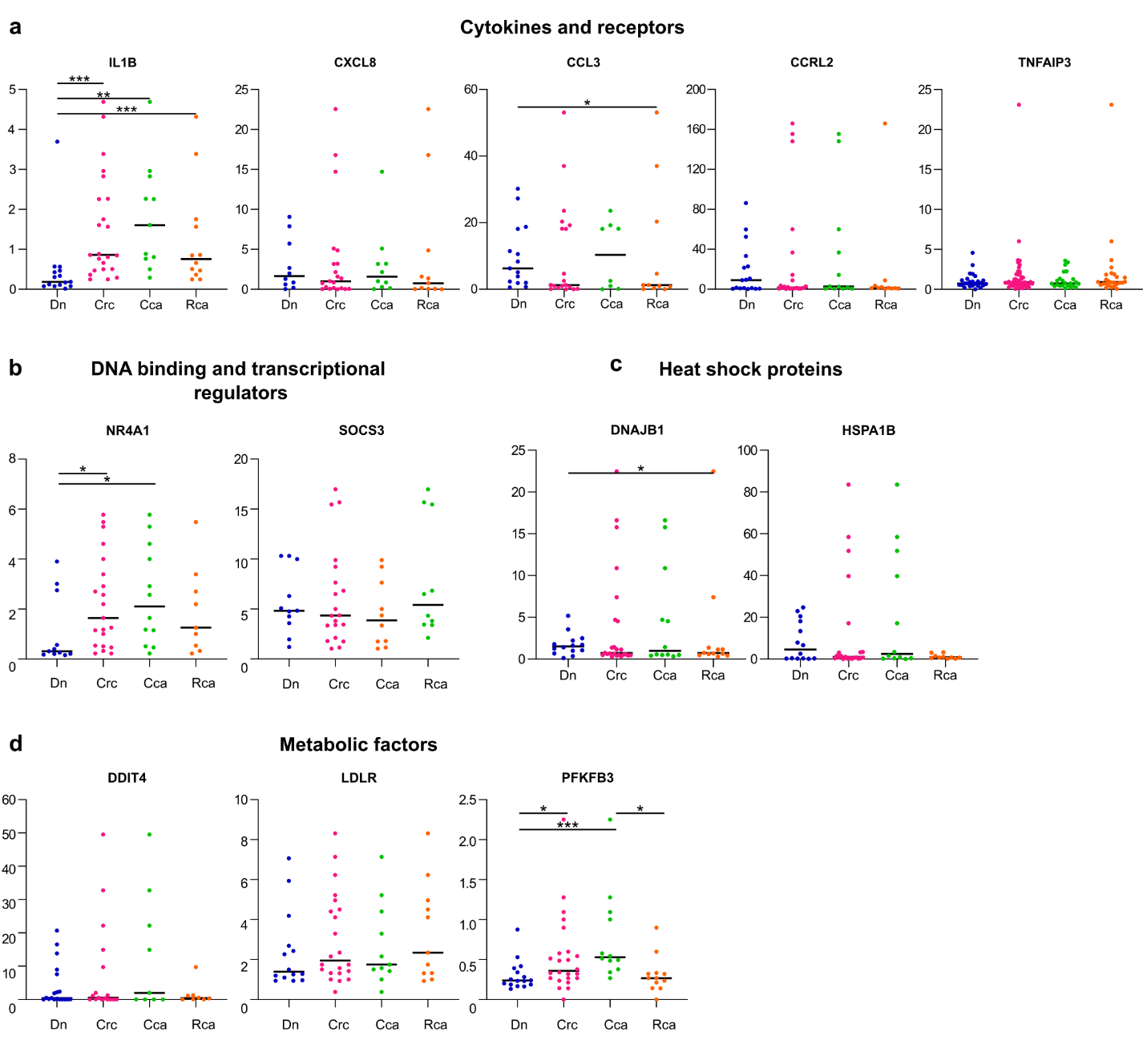


**Supplemental Figure S5**. **Validation of RNAseq results for CRC patients and healthy donors**. **a-d**, Real-time PCR analysis was performed for the comparison of the expression of validation gene set in colorectal cancer patents (Crc), colon cancer patients (Cca) and rectal cancer patients (Rca) before any treatment and in healthy individuals (Dn). Genes were functionally attributed to cytokines and receptors (**a**), DNA binding and transcriptional regulators (**b**), heat shock proteins (**c**) and metabolic factors (**d**). * - p<0,05, ** - p<0,01, *** - p<0,001.
