# Supplementary material for: PFKFB3 overexpression in monocytes of patients with colon but not rectal cancer programs pro-tumor macrophages and is indicative for higher risk of tumor relapse
Source: Front Immunol. 2023 Jan 17;13:1080501. doi: 10.3389/fimmu.2022.1080501 (PMC9887047; doi:10.3389/fimmu.2022.1080501)
Supplement: Supplementary file 2 [file Table_1.docx]

**Supplemental Table S1** Sequences of primers with original design used for quantitative real-time PCR analysis

| Gene | Amplicon | Sequence |
| --- | --- | --- |
| GAPDH  NM_002046.7 | 124 bp | F 5'- GCCAGCCGAGCCACATC-3' |
|  |  | R 5'- GGCAACAATATCCACTTTACCAGA-3' |
| DDIT4  NM_019058.4 | 166 bp | F 5'- GTGTCAGGGATCACTTGGGA-3' |
|  |  | R 5'- AGATGGAAGACCCAGATGGC-3' |
| CCRL2  NM_003965.5 | 81 bp | F 5'- GGCACCAGAGGATGAATATGATG-3' |
|  |  | R 5'- GCGTCATACTTGTCACATTGC-3' |
| CCL3  NM_002983.3 | 81 bp | F 5'- ACGGGCAGCAGACAGTG-3' |
|  |  | R 5'- GCATGATTCTGAGCAGGTGAC-3' |
| PFKFB3  NM_001282630.3 | 117 bp | F 5'- ACAAATGCGACAGGGACTTG-3' |
|  |  | R 5'- GGATGTTCATCAGGTAGTACACG-3' |
| TNFAIP3  NM_006290.4 | 80 bp | F 5'- CAGGACTTGGGACTTTGCG-3' |
|  |  | R 5'- TGAGGAAGGACTTGTTCAGC-3' |
| SOCS3  NM_001378933.1 | 103 bp | F 5'- GATTCTACTCTGTGCCTCCTGA-3' |
|  |  | R 5'- GCTGAGTATGTGGCTTTCCTATG-3' |
| LDLR  NM_000527.5 | 101 bp | F 5'- AAGAAGCCCAGTAGCGTGA-3' |
|  |  | R 5'- AGCCGCCAGTTCTTCCATA-3' |
| DNAJB1  NM_006145.3 | 99 bp | F 5'- CCCACATCCACCTCCAGTC-3' |
|  |  | R 5'- GACATTATCTACCAGCCAGAAGC-3' |
| HES4  NM_021170.4 | 76 bp | F 5'- CGCCCTCAGAAAAGAGAGC-3' |
|  |  | R 5'- CAGGTGTCTCACGGTCATCT-3' |
| IL1B  NM_000576.3 | 147 bp | F 5'- CTCTTCAGCCAATCTTCATTGC-3' |
|  |  | R 5'- AGGAGCACTTCATCTGTTTAGG-3' |
| NR4A1  NM_002135.5 | 109 bp | F 5'- ATGGTGAAGGAAGTTGTCCGA-3' |
|  |  | R 5'- GGGAAGTGAGGAGATTGGCA-3' |
| HSPA1B  NM_005346.6 | 77 bp | F 5'- AGTATGTTTGTCTTTGAGGTGGAC-3' |
|  |  | R 5'- GCTGAAGCAGAAATGACATAGGA-3' |
| CXCL8  NM_000584.4 | 150 bp | F 5'- CACTGTGTGTAAACATGACTTCC -3' |
|  |  | R 5'- GGTGGAAAGGTTTGGAGTATGT -3' |

Notes: all probes – FAM →BHQ1; NM – number of RNA sequence in NCBI Nucleotide Database (<http://www.ncbi.nlm.nih.gov/nuccore>); bp – base pair; F – forward primer; R – reverse primer. All primers have the original design.

**Supplemental Table S2** Clinical-pathological parameters of patients with colorectal cancer from TCGA cohort

| Clinical-pathological parameters | Colorectal cancer  (N=417) | Colon cancer  (N=305, 73,1%) | Rectal cancer  (N=112, 26,9%) |
| --- | --- | --- | --- |
| Age | 66,4±12,7 | 67,2±13 | 64,2±11,6 |
| Sex  Male  Female | N=217, 52%  N=200, 48% | N=160, 52,5%  N=145, 47,5% | N=57, 50,9%  N=55, 49,1% |
| Stage  I-II (early)  III (advanced) | N=271, 65%  N=146, 35% | N=201, 65,9%  N=104, 43,1% | N=69, 61,6%  N=43, 48,4% |
| T1-2  T3-4 | N=106, 25,4%  N=311, 74,6% | N=76, 24,9%  N=229, 75,1% | N=27, 24,1%  N=85, 75,9% |
| Recurrence  Yes  No  Unknown | N=68, 16,3%  N=327, 78,4%  N=22, 5,3% | N=52, 17%  N=236, 77,4%  N=17, 5,6% | N=16, 14,3%  N=91, 81,3%  N=5, 4,4% |

**Supplemental Table S3** Clinical and pathological parameters of patients involved in flow cytometry study

|  | Colon cancer (N) | Rectal cancer (N) |
| --- | --- | --- |
| Age | 66,3±7,9 (34) | 63,7±8,4 (36) |
| Gender | Male (13)  Female (21) | Male (22)  Female (14) |
| Tumor size | T_2_ (2)  T_3_ (14)  T_4_ (14) | T_2_ (5)  T_3_ (15)  T_4_ (16) |
| Lymph node status | N_0_ (17)  N_1-3_ (11) | N_0_ (18)  N_1-3_ (13) |
| Hematogenous  metastasis | M_0_ (27)  M_1_ (4) | M_0_ (30)  M_1_ (4) |
| NAC response | Not applicable | TRG_1-2_ (4)  TRG_3-5_ (16) |

Notes: Five-grade Mandard Tumor Regression Grading (TRG) system was used for assessment of NAC response in rectal cancer patients. TRG_1-2_ were defined as good response, and TRG_3-5_ – bad response.
